# GraPES: The Granule Protein Enrichment Server for prediction of biological condensate constituents

**DOI:** 10.1093/nar/gkac279

**Published:** 2022-04-26

**Authors:** Erich R Kuechler, Matthew Jacobson, Thibault Mayor, Jörg Gsponer

**Affiliations:** Department of Biochemistry and Molecular Biology, Michael Smith Laboratories, University of British Columbia, Vancouver, BC, Canada; Department of Biochemistry and Molecular Biology, Michael Smith Laboratories, University of British Columbia, Vancouver, BC, Canada; Department of Biochemistry and Molecular Biology, Michael Smith Laboratories, University of British Columbia, Vancouver, BC, Canada; Department of Biochemistry and Molecular Biology, Michael Smith Laboratories, University of British Columbia, Vancouver, BC, Canada

## Abstract

Phase separation-based condensate formation is a novel working paradigm in biology, helping to rationalize many important cellular phenomena including the assembly of membraneless organelles. Uncovering the functional impact of cellular condensates requires a better knowledge of these condensates’ constituents. Herein, we introduce the webserver GraPES (Granule Protein Enrichment Server), a user-friendly online interface containing the MaGS and MaGSeq predictors, which provide propensity scores for proteins’ localization into cellular condensates. Our webpage contains models trained on human (*Homo sapiens*) and yeast (*Saccharomyces cerevisiae*) stress granule proteins. MaGS utilizes experimentally-based protein features for prediction, whereas MaGSeq is an entirely protein sequence-based implementation. GraPES is implemented in HTML/CSS and Javascript and is freely available for public use at https://grapes.msl.ubc.ca/. Documentation for using the provided webtools, descriptions of their methodology, and implementation notes can be found on the webpage.

## Graphical Abstract

**Graphical Abstract:**
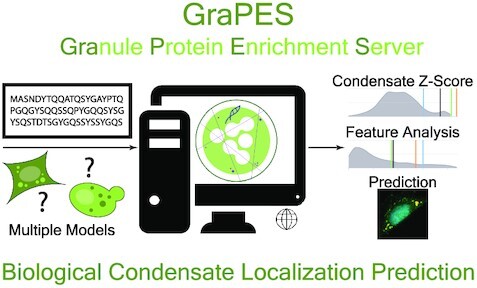
GraPES is a user-friendly online interface which provides propensity scores for proteins’ localization into cellular condensates with feature analysis and graphical outputs.

## INTRODUCTION

Protein phase separation has been thrust to the forefront of molecular biology over the past decade (1–3). During protein condensate formation, driver biopolymers are thought to seed the formation of protein-rich foci within the cellular milieu (4,5). Stress granules (SGs) are a specific type of biological condensate which can be induced to form by glucose starvation (6), viral infection (7), or other external stresses such as temperature shock (8,9). SGs and other related membraneless organelles are of keen interest to the biomolecular research community for being linked to both long-term cell viability and a variety of protein aggregation-based diseases (10–12). Previously, we found that proteins within curated SGs sets are more disordered, soluble, and abundant as well as containing more annotated post-translational modifications than cytosolic proteins in general and the proteome as a whole. Furthermore, SG proteins were found to have multiple ordered domains, a large number of protein-protein interactions, and to interact frequently with RNA (13). Our findings were consistent with the hypothesis that SGs are liquid phase-separated compartments and that proteins which readily enter these assemblies are likely resting near their solubility limits to aid the cell in its capacity to rapidly mobilize proteins into SGs.

Based on our findings and the availability of well-curated SG sets, we designed two predictors to score the propensity of proteins to localize to SGs. Ample testing revealed that these prediction scores were elevated for SG constituent proteins but also for those proteins which localize into other biological condensates. This finding was somewhat expected, given the biophysical mechanism of phase separation that is thought to underly condensate formation. Thus, by exploiting publicly available proteomic and database information, we introduced these two generalized predictors for protein localization into biological condensates, one for mammalian cells and one for yeast cells, called MaGS (rebranded here as the Membraneless organelle and Granule Score to account for both the mammalian and yeast predictors). These tools provide some of the highest known confidence predictions for biological condensate localization, outperforming similar computational methods (13).

Herein, we present a user-friendly, web-based interface called GraPES (Granule Protein Enrichment Server), which houses the original MaGS as well as the newly developed and complementary MaGSeq models. On the GraPES website (https://grapes.msl.ubc.ca/) users can look up a variety of pre-calculated propensity MaGS values for human and yeast proteins or obtain novel MaGSeq predictions from FASTA formatted protein sequences which, while these models have been optimized for mammalian or yeast condensate predictions, can in principle be used for any eukaryotic organism.

## MATERIALS AND METHODS

GraPES includes the two predictors: MaGS and MaGSeq. MaGS has been benchmarked and predictions validated experimentally previously (13). In short, it is based on a general linearized model (GLM) that uses the protein features of protein abundance (14), percent protein intrinsic disorder (15), number of annotated phosphorylation sites (16,17), PScore (18), Camsol score (19), RNA interaction (20,21), and percent composition of leucine and glycine to generate predictions for protein localization into biological condensates. Based on MaGS predictions, we were able to experimentally confirm, upon arsenite stress, SG localization of two highly scored proteins that were previously unknown to locate into condensates. However, a number of features used by MaGS are experimentally measured which, due to the lack of complete experimental data, limits the application range of this model. Wanting to expand the scope of our predictor, we have now complemented MaGS with MaGSeq, a new model that provides similar analysis utilizing sequence-based features only.

### Datasets

To avoid over training our models on homologous proteins, we first clustered proteins based on sequence similarity using CD-Hit at the 35% sequence homology level resulting in a total of 13280 non-homologous proteins for human and 4465 proteins for yeast (22). We then created the positive SG sets for training and validation by matching these non-homologous proteins with human and yeast SG proteins that we had previously assembled from high-confidence mass spectrometry and colocalization immuno-fluorescence studies (13,23–28). We divided these stress granule positive protein sets (388 human and 301 yeast proteins) into two-thirds for training and one-third for validation. Additionally, we created fully-independent test sets of positive stress granule proteins for performance evaluation and comparison: 131 human proteins were gathered from a stress granule database (29) and 116 yeast proteins from the drLLPS database (30). Importantly, these test sets do not contain any training or validation proteins and have less than 35% sequence identity with any of these proteins. The non-homologous human and yeast proteins that are not part of the positive training, validation or test sets were used to generate balanced negative sets (Supplementary Table S1).

Data sets for condensate-specific comparisons for P-bodies, Cajal bodies, Nucleolus, PML-bodies, Nuclear speckles, Centrosome-Spindle pole bodies, and *in vitro* condensates were constructed from the drLLPS database, using proteins from the ‘Scaffold’ and Client’ classifications (30). Additional data sets for comparison of model performance in different organisms were collected from the drLLPS database for nematode (*C. elegans*), mouse (*M. musculus*), fruit fly (*D. melanogaster*), and thale cress (*A. thaliana*). Proteins were taken from the ‘Scaffold’ and ‘Client’ classifications of the Cajal body, P-body, Stress granule, U-body, PcG body, Nuclear speckle, Nucleolus, and proteins in the ‘Other’ groups, where applicable, to construct ‘condensate protein’ data sets. Size-balanced negative controls were then constructed using randomly selected proteins not included in the drLLPS database.

### Model parameterization

**Figure 1.**
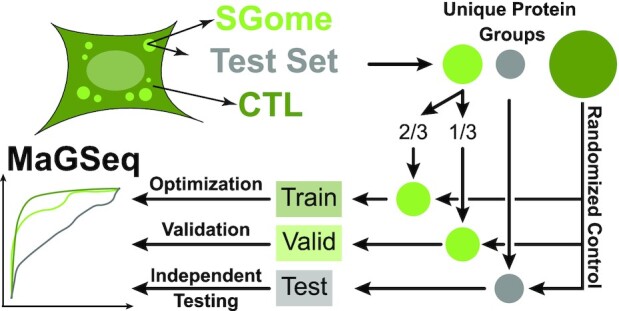
The general workflow of the MaGSeq parameterization. SGome positive SG proteins are divided 2/3 for training and 1/3 for validation. Once the general linearized models were optimized, they were then evaluated on an independent test set and compared to other granule and phase separation prediction software.

We parameterized a general linearized model (GLM) for MaGSeq by following a standard protocol in which the model is optimized using training and validation sets and then benchmarked against a fully independent test set (Figure 1). We initially assessed protein features in the SG sets (excluding the test sets) with a linear discriminant analysis (LDA) in order to see which features best separate positive and negative hits. We used the GLM package in R to generate the general linearized model (R Core Team (2020). R: A language and environment for statistical computing. R Foundation for Statistical Computing, Vienna, Austria. https://www.R-project.org/). We then removed features systematically and assessed model performance using Areas Under Curve (AUC) values of the Receiver Operating Characteristics (ROC) functions on the balanced positive and negative validation sets while monitoring feature contributions to the GLM fit. The features selected for the optimized MaGSeq models include: percent protein intrinsic disorder (15), π-π interaction PScore (18), Soluprot protein solubility score (31), RNA-binding interaction RBPscore (32), GRAVY protein hydrophobicity score (33), and the total composition of charged amino acids as well as specific amino acids (D, A, V, I, M, F) for human, and the TANGO score (34), Soluprot score (31), total sequence length as well as percent composition of some specific acids (S, A, P) for yeast. This difference in features used for the human and yeast models is consistent with the differing viscoelastic properties of the stress granules observed between the systems. In mammalian cells these granules appear very liquid-like, while in yeast these granules do not (35); accordingly, several yeast stress granule proteins that we assessed display little fluorescence recovery after photobleaching (27). Protein intrinsic disorder, π-π, solubility, and RNA-binding scores were calculated using the respective computational platforms, while the remaining features were calculated with in-house Perl scripts (36). After the optimization of the models, we generated scores for all non-homologous proteins in the clustered proteomes, which we then used to generate Z-scores for each protein as the final output of the model. Finally, we used the independent test sets for benchmarking and comparisons with established granule and protein phase separation predictors as well as with MaGS, which had not seen the test set proteins during its training either. To facilitate prediction interpretation, we estimated cutoff values by approximating the model worthiness (NCSS 2021 Statistical Software (2021), NCSS, LLC. Kaysville, Utah, USA, ncss.com/software/ncss). We determined model specificity at the balance point of ROC curves for a moderate threshold. High and low threshold values were estimated by bisecting the specificity on either side of the moderate threshold (Supplementary Figure S1). The selected cutoffs are provided below and are available for users on the homepage of GraPES.

## RESULTS

### MaGSeq performance

**Figure 2.**
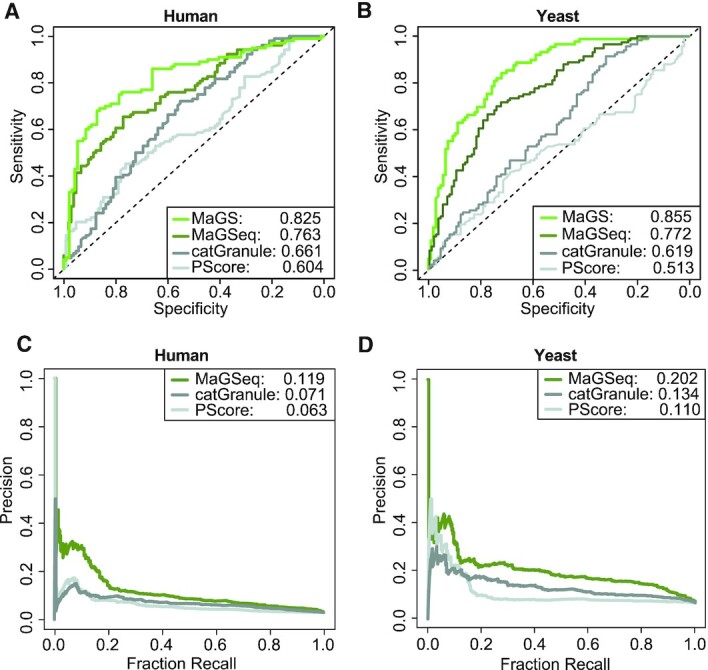
Performance comparison. AUC plots of the MaGS and MaGSeq models for human (**A)** and yeast **(B)** parameterizations as compared to the PScore (18) and catGranule (37) models. Additionally, PR curves for the MaGSeq model for human **(C)** and yeast **(D)** parameterizations as compared to PScore and catGranule.

During parameter optimization, the MaGSeq models reached AUC values of 0.78 and 0.79 on the human and yeast validation sets, respectively (Supplementary Figure S2). More importantly, with AUCs of 0.76 and 0.77 for human and yeast test set proteins, respectively, MaGSeq consistently outperforms the current state-of the-art sequence-based methods PScore (18) and catGranule (37) (Figure 2A-B). It needs to be stressed that catGranule was specifically designed to predict granule proteins, while the PScore calculates the probable amount of π-π interactions in protein sequences and, therefore, is a more generic predictor of a protein's likelihood to phase separate *in vitro*. However, it is thought that proteins which can form protein droplets *in vitro* are likely to act as ‘drivers’ in the formation of biological condensates within the cell. As the number of proteins that do not localize to SGs is significantly larger than the ones that do, we also calculated precision-recall (PR) curves using the complete negative test set not used in training or validation. Examination of these PR curves reveals that the MaGSeq models have a higher precision than the other methods at almost all levels of sensitivity (Figure 2C-D). We previously showed that, although we parameterized the MaGS models using SG proteins only, MaGS also predict localization of proteins in other condensates (13). We verified that MaGSeq models also show significantly higher scores for proteins in other condensates with the exception of the centrosome/spindle and promyelocytic leukaemia (PML) bodies (Supplementary Figure S3). Moreover, MaGSeq models show significantly higher scores for proteins known to be part of condensates in the organisms *C. elegans*, *M. musculus*, *D. melanogaster*, and *A. thaliana* when compared to randomized controls (Supplementary Figure S4).

### Websever description

**Figure 3.**
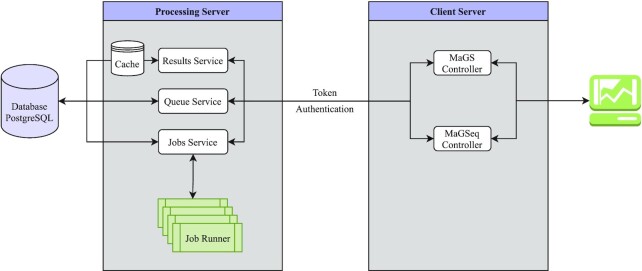
The general workflow of the GraPES server. The user can lookup precalculated MaGS information or, alternately, run their own novel predictions on the web server. The GRaPES system is separated into a Processing Server and Client Server. The user's browser interacts with the Client Server to request results and submit jobs under their session identifier. The Client Server then forwards these requests alongside an authentication token to the Processing Server via HTTP calls. The Processing Server responds to requests for 1) results by retrieving them from the associated database or cache, 2) queue information by considering the state of pending and completed jobs, and 3) job submissions by storing the necessary information for future processing. MaGSeq jobs are scheduled to Job Runners in approximate order of submission while attempting to prevent single users from monopolizing the queue. Proteomic background distributions and their associated kernel density estimates of protein features are precalculated and cached in memory.

GraPES is a HTML/CSS and Javascript webserver that houses four separate biological condensate protein localization prediction tools: the two MaGS and MaGSeq predictors, each with one parameterization for *Homo sapiens* and one for *Saccharomyces cerevisiae*. The general workflow of the server can be seen in Figure 3. The user is initially located on the homepage, where the user can choose between the MaGS and MaGSeq models. The pre-calculated MaGS database contains predictions for 16947 human and 4883 yeast proteins. The two MaGSeq themed predictors are able to take any protein sequence comprised of the canonical 20 amino acids. For these MaGSeq predictors, inputs are passed into a BASH environment where the required calculations of protein features are completed on computational clusters on the server's end. The output files from these programs are then parsed and tabulated using in-house Perl scripts and passed to the R software package which houses the optimized GLM models. Calculated scores are then passed back to the webserver, which will then generate outputs and present them to the user. Users can submit any number of protein sequences at the same time, but each sequence will be submitted into the queuing system, and run separately. Completed jobs will be held on the server for a minimum of 14 days. Due to the computationally intensive BLAST alignment required in the disorder calculation, predictions can take on the order of half an hour to an hour depending on sequence length. Thus, email notifications are recommended. However, any prediction calculation that is queued and contains an identical sequence to one that is currently stored on the server will access the cached results, and the user will not have to wait for prediction calculations.

### Input

Precalculated MaGS values can be searched using either a protein's UniProt accession number or with the gene name. MaGSeq predictors take a FASTA formatted protein sequence with a minimal primary sequence length of 150 amino acids, a residue limitation due to some of the software used in the prediction of protein features used in the model. An example sequence, showing the correct format, is provided.

### Output

For any query protein, the server provides the prediction Z-score as well as the feature scores used to generate the predictions. In addition, distributions of precomputed Z-scores of the human and yeast proteomes are provided for comparison, as well as the proteomic distributions of each protein feature used in score computation. As reference, precomputed Z-scores and feature scores of the known biological condensate markers PAB1, G3BP1, DCP1, and DCP2 are shown. Outputs are available as graphical plots and in numerical form. This information can be downloaded as images and/or as plain text in either CSV or JSON format.

### Usage example

Landing on the GraPES server, the user is first located on the homepage which gives access to both MaGS and MaGSeq prediction models. It is recommended that users obtain predictions from the MaGS models first, as they are more accurate methods. If a score is available, the user can reference the suggested cutoffs to interpret whether the MaGS Z-score obtained represents a high (>1.16 for human and > 1.08 for yeast), moderate (between 1.16 and 0.66 for human and between 1.08 and 0.58 for yeast) or low propensity (between 0.66 and −0.36 for human and between 0.58 and −0.39 for yeast) for cellular condensate inclusion. However, if no score is available, because experimental features used by MaGS are missing for the query protein, then MaGSeq can be used. It should be noted that scores from MaGS and MaGSeq are not directly comparable. Therefore, we provide different cutoff values for MaGSeq. Specifically, proteins with high, moderate and low propensity for cellular condensate localization are those human (yeast) proteins with scores > 0.90 (>0.89), between 0.90 and 0.56 (0.89 and 0.25), and between 0.56 and −0.33 (0.25 and −0.45), respectively.

**Figure 4.**
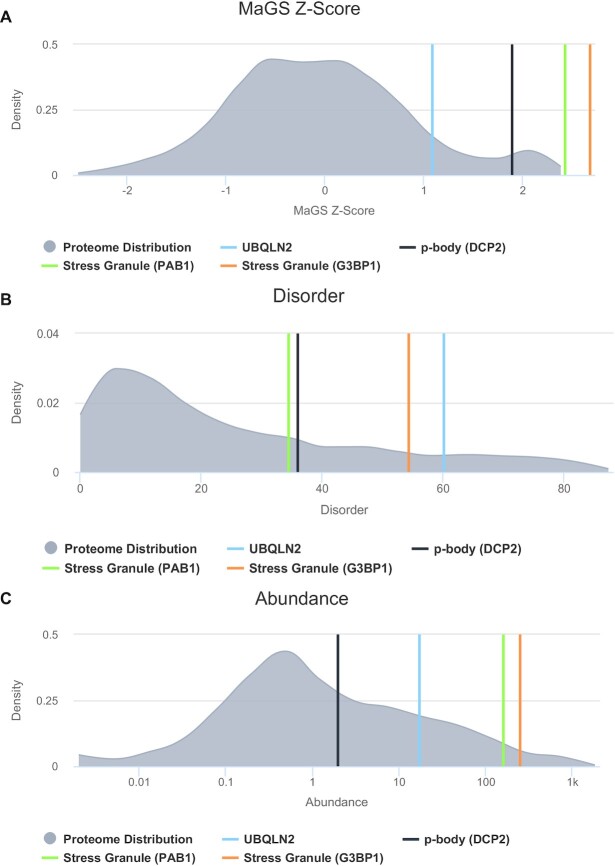
Example MaGS Z-score prediction and protein feature plots for the UBQLN2 protein. Shown is the predicted MaGS Z-score**(A)**, the percentage of intrinsic disorder **(B)**, and the protein abundance **(C)** of UBQLN2. The proteomic background distribution is shown in gray, while the protein of interest is marked in the distribution by the light blue line. Markers for SGs and p-bodies are also provided to help the user gauge the relative level of that feature as compared to known condensate constituents.

As a specific usage example, we show the results for Ubiquilin-2 (UBQLN2), a protein in our independent test set. It receives a MaGS score of 1.08, indicating that it has a moderate to high propensity to be associated with biological condensates, and indeed it has been noted to phase separate in a number of different conditions (38) and has been seen in stress granules (39) and other ALS/FTD-linked complexes (40). Users can see where this score falls in the distribution of all predicted Z-scores (Figure 4A) as well as how this Z-score compares to known biological condensate markers. Here the proteomic distribution is shown as a grey kernel density estimation distribution and biological condensate markers PAB1, G3BP1, DCP1, and DCP2 are displayed in different colours. The query protein, UBQLN2 in this case, is always in light blue. Furthermore, plots of the protein features used in the model are provided. For UBQLN2, examples of percent protein disorder (Figure 4B) and protein abundance (Figure 4C) can be examined for further insight. We and others have previously shown (13,41) that proteins in SGs are enriched for percent disorder, likely to allow for the formation of multivalent interactions (13). Moreover, SG proteins often have an elevated abundance, which may allow these proteins to remain close to their saturation concentrations (13). UBQLN2 ranks highly in both of these metrics as compared to the background distribution, and places among or close to many of the condensate markers. Examining the feature plots will help in the design of variants that may enhance or reduce propensity for granule localization. Importantly, plots are interactive and users can zoom in on a given range on the plot by clicking and dragging over the desired values. Images of the plots can be downloaded in a variety of formats from the dropdown menu located on the upper right of the plot.

A protein that is not contained in the MaGS database is LINE-1. This protein has recently been found to phase separate through its N-terminus and coiled-coil domain (42) and is associated with stress granules and other cellular condensates (43). When examined with MaGSeq, this protein obtains a Z-score of 0.9804, placing it in the ‘high propensity’ range for condensate localization. Thus, MaGSeq extends the application range of our approach. However, it is important to keep in mind that cellular condensate formation is a complex phenomenon and competing factors could limit the applicability of a purely sequence-based approach.

## FINAL REMARKS

The performance of MaGS is, as expected, higher than that of MaGSeq due to the model's use of experimental data. However, MaGS can only be applied to proteins for which protein abundance and annotated phosphorylation sites are known, while the MaGSeq models only use sequence information, thus providing flexibility to potentially query splice variants as well as protein sequences of other species. Indeed, MaGSeq predictors show significantly higher scores for known condensate proteins over randomized controls for a number of organisms (Supplementary Figure S4). A closer look at these results suggests that the human parameterization of MaGSeq is more appropriate for the prediction of condensate proteins in mice and nematode, while the yeast parametrization would be better for plants. While MaGSeq scores for condensate proteins in flies are significantly higher than controls, the scores are less discriminative than in other organisms. In any case, it needs to be stressed that scores and thresholds are not optimized for organisms other than human or yeast and, therefore, care should be taken when evaluating a protein's prediction score.

Additionally, while the MaGS and MaGSeq models were parameterized to predict stress granule proteins, these models identify proteins associated with many other biological condensates (Supplementary Figure S3). This aspect extends the use of these models to a wide variety of membraneless organelles but also demands that the user critically assess their proteins after a score is obtained. For instance, histone proteins obtain high MaGS scores and are unlikely to be found within stress granules due to their biological context. However, it has been recently found that these proteins do undergo phase separation in the nuclei of HeLa cells (44), and the physicochemical properties that lead to this behavior are likely similar to those which drive proteins into granules.

MaGS and MaGSeq are complementary because they use different features to assess condensate localization and have different application ranges. Scores cannot be directly compared between the two models; however, more confidence can be gained if a protein obtains high scores across both models. If the models disagree in their predictions, then more weight should be given to the MaGS predictors as they are more accurate and account for biological features that go beyond what the primary sequence can provide. For instance, the protein Nab6 in *S. cerevisiae* is known to localize to stress granules and obtains a MaGS of 1.41 and a MaGSeq of 0.64, having a ‘high’ propensity in MaGS and a ‘moderate’ propensity in MaGSeq.

In the analyses and validation of MaGS predictions (13), we found that several proteins receive high scores that have not yet been found to belong to known cellular condensates. There could be several explanations for these results; it is likely that some of these proteins simply remained undetected by current mass spectrometry techniques, or the proteins contain features that are identical to those of known condensate proteins, but these proteins are contained within a complex or have interactions that prevent condensate localization. The aforementioned histone proteins follow the latter explanation. Even with these limitations, the MaGS models can help provide insight into biological condensates and protein phase separation.

## DATA AVAILABILITY

GraPES is an open-source web application designed to increase the usability and application of the MaGS and MaGSeq methods for the prediction of protein localization into biological condensates. This platform is deployed at https://grapes.msl.ubc.ca/, the website source code is provided on Github https://github.com/JacobsonMT/GraPES, and source code for the GLM models and model data can be found at https://github.com/ekuec/2020_grapes_server for advanced users or those who wish to do high-throughput calculations using the MaGS or MaGSeq models.

## Supplementary Material

gkac279_Supplemental_Files
