# Supplementary material for: GraPES: The Granule Protein Enrichment Server for prediction of biological condensate constituents
Source: Nucleic Acids Res. 2022 Apr 26;50(W1):W384–91. doi: 10.1093/nar/gkac279 (PMC9252806; doi:10.1093/nar/gkac279)
Supplement: gkac279_Supplemental_Files [file gkac279_supplemental_files.zip › 2021_NAR_Supplement_combined.pdf]

Figure S1

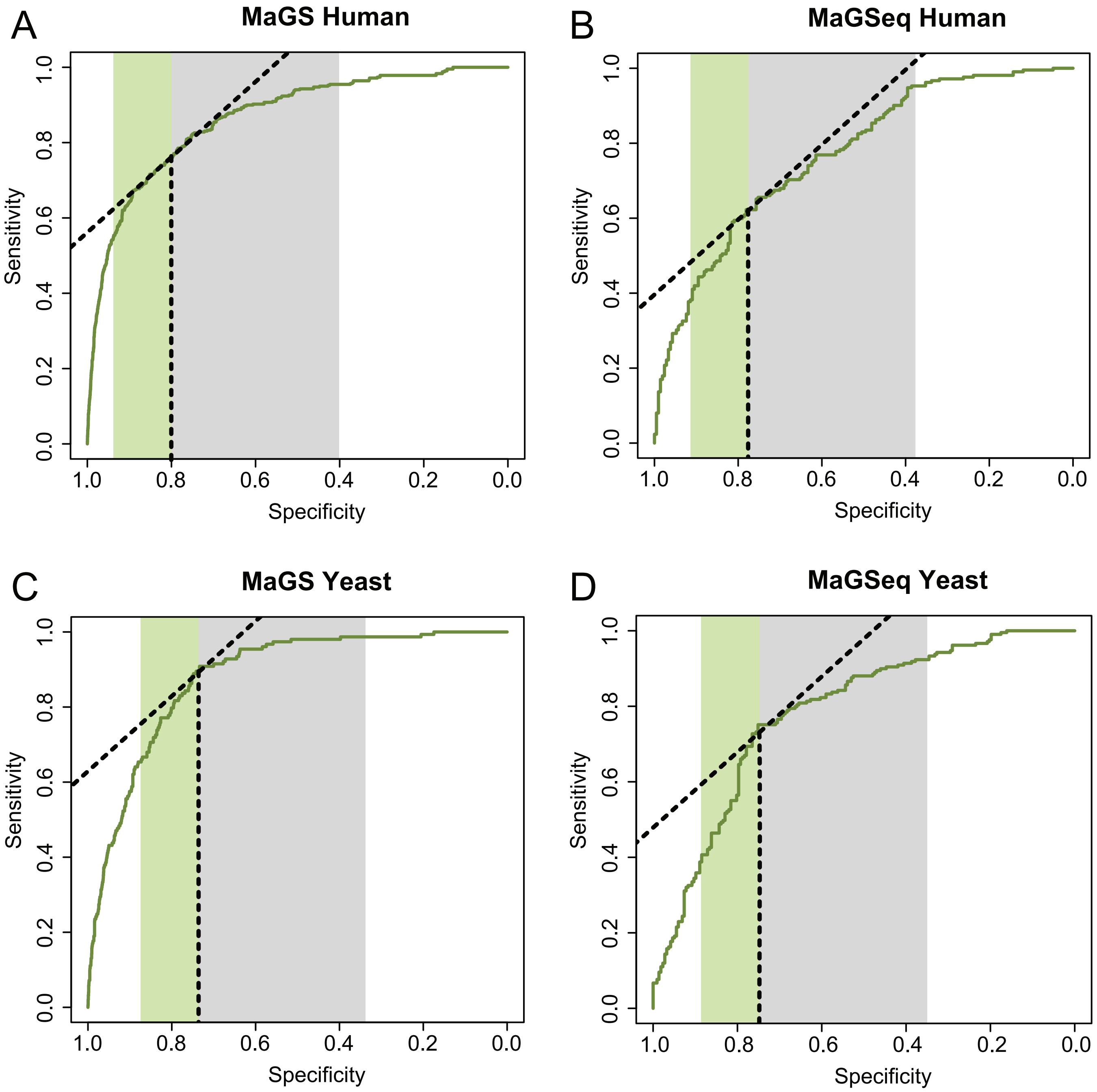

Figure S1: Determination of suggested cutoff values. We approximated the model worthiness by determining model specificity at the balance point of ROC curves for human MaGS (A) and MaGSeq (B) as well as yeast MaGS (C) and MaGSeq (D). High and low cutoff ranges are indicated by the ends of the light green and grey boxes, respectively.

Figure S2

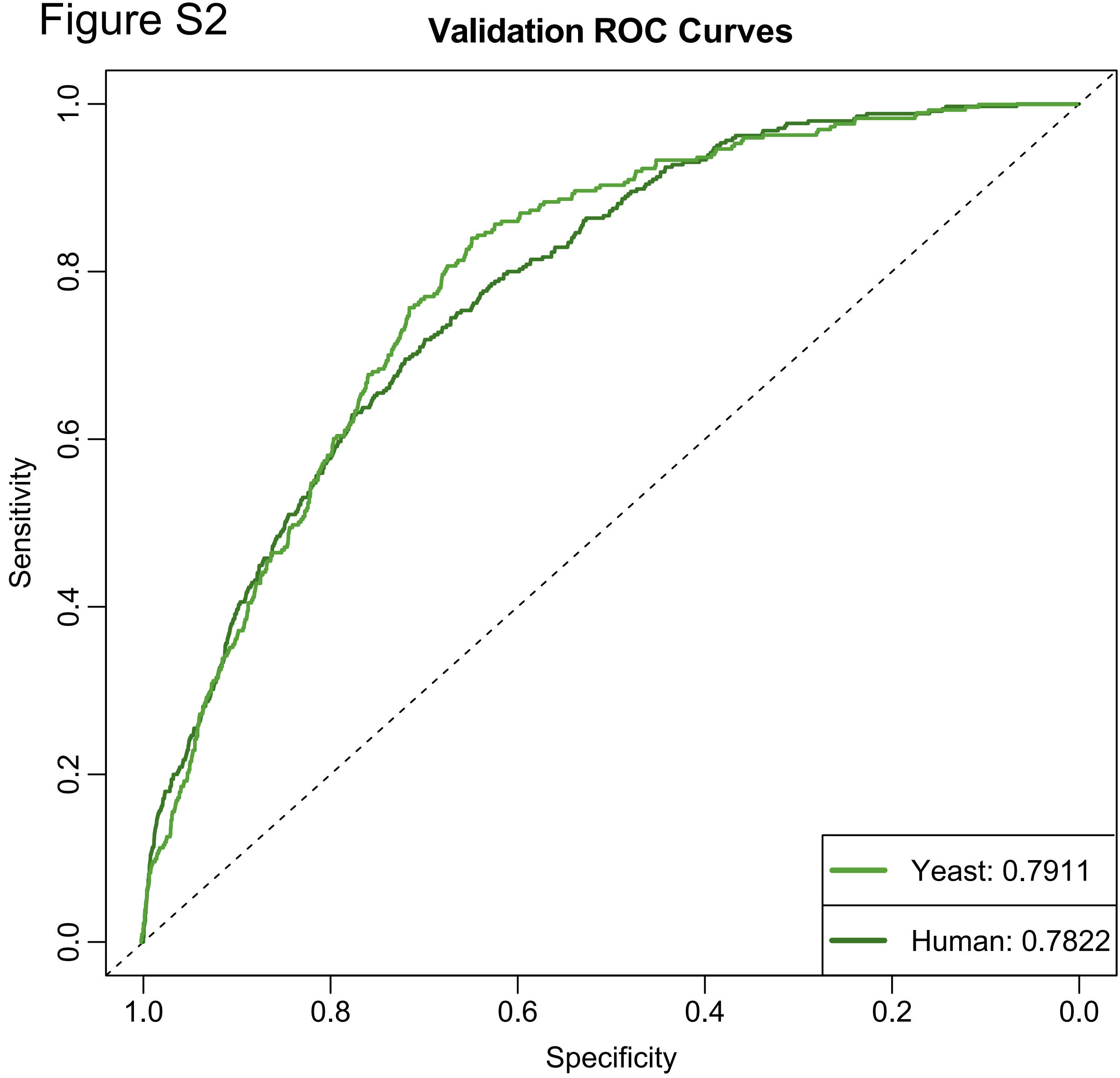

Figure S2: Validation curves for the training of the human and yeast MaGSeq models.

Figure S3

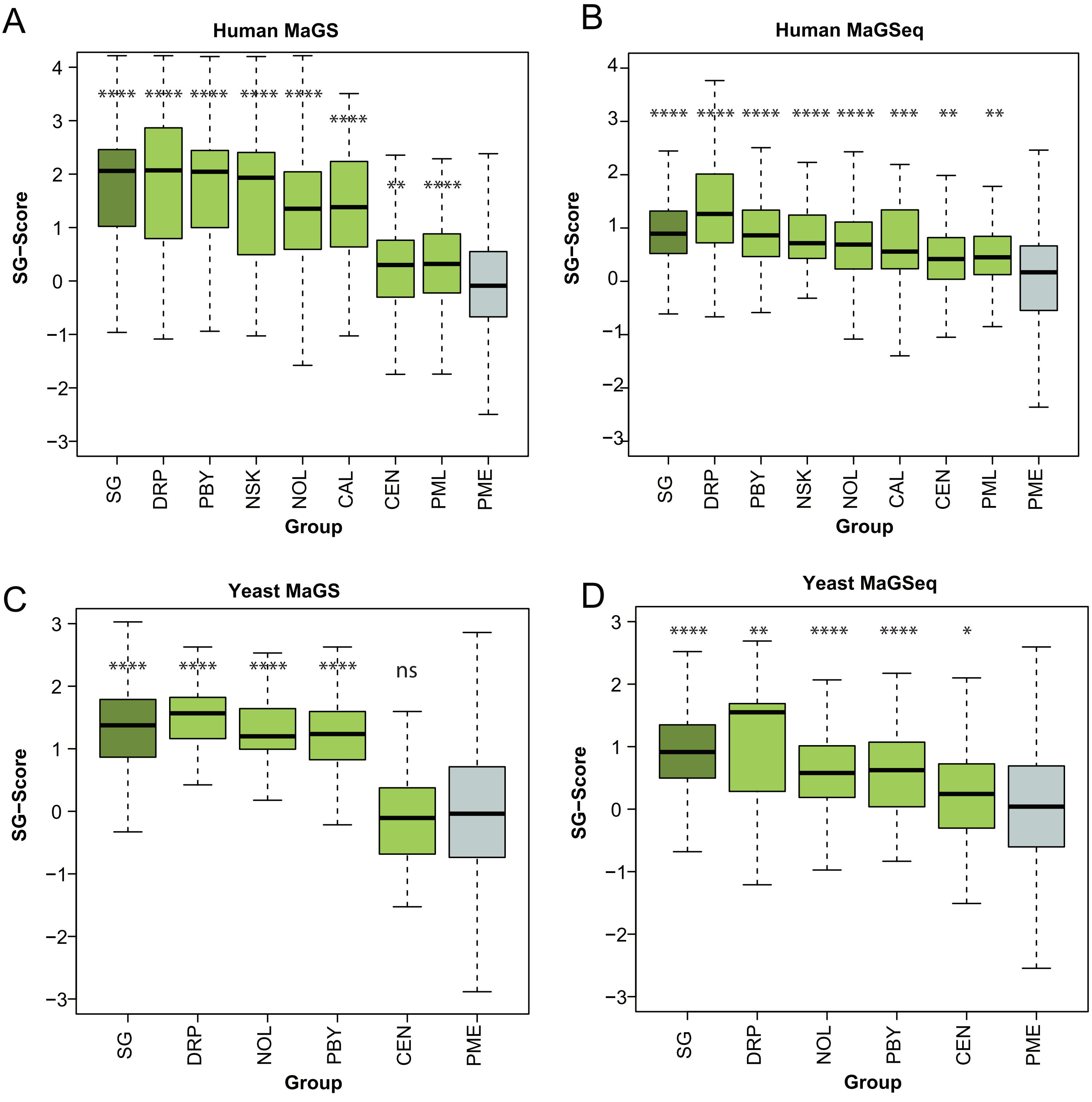

Figure S3: Distributions of MaGS and MaGSeq scores for different cellular condensates, for human (A-B) and yeast (C-D) parametrizations. We compare scores for our stress granule set (SG, n=457/321) to those of proteins found in *in vitro* condensates or droplet (DRP, n=81/15), p-bodies (PBY, n=264/58), nuclear speckles (NSK, n=84/NA), the nucleolus (NOL, n=1108/114), Cajal bodies, (CAL, n=32/NA), centrosome-spindle bodies (CEN, n=510/87), and PML bodies (PML, n=65/NA) (n=human/yeast). MaGS predictor scores separate proteins localizing to many types of biological condensates from their respective proteomes, however cannot distinguish well between these condensates. Scores for all condensates proteins, except for those centrosome-spindle body and PML bodies, are significantly higher than the reference proteome scores (p-value< 1e-2, Wilcoxson test). Stars indicate significance: **** for <1e-5, *** for < 1d-4, ** for <1e-3, and * for < 1e-2. Nonsignificant differences are marked with 'ns'.

Figure S4

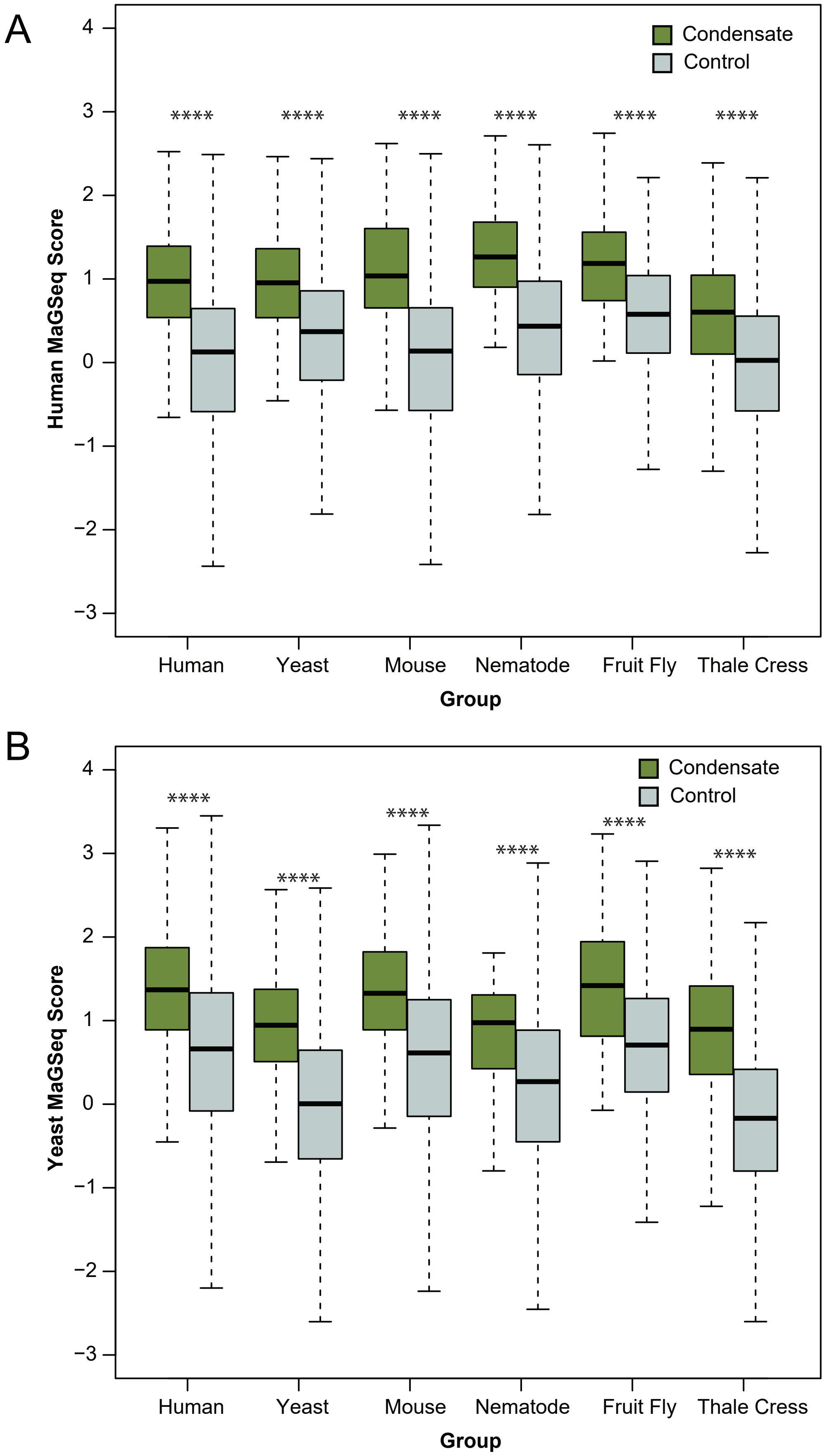

Figure S4: Prediction scores for proteins in condensates of other organisms. Nematode (*C. elegans,* n=58), mouse (*M. musculus,* n=62), fruit fly (*D. melanogaster,* n=41), and thale cress (*A. thaliana,* n=1903) proteins were taken from the 'Scaffold' and 'Client' classifications of the Cajal body, Droplet, P-body, Stress granule, U-body, PcG body, Nuclear speckle, Nucleolus, and others condensate types of the drLLPS database. Results from the human (A) and yeast (B) parameterizations of MaGSeq are shown. All condensate groups have significantly higher scores than their size-balanced negative controls (p-value<1e-5, Wilcoxson test) indicated by stars (****).
